# A mutual titer-enhancing relationship and similar localization patterns between *Citrus exocortis viroid* and *Hop stunt viroid* co-infecting two citrus cultivars

**DOI:** 10.1186/s12985-015-0357-6

**Published:** 2015-09-17

**Authors:** Chun-Yi Lin, Meng-Ling Wu, Tang-Long Shen, Ting-Hsuan Hung

**Affiliations:** Department of Plant Pathology and Microbiology, National Taiwan University, Taipei, 10617 Taiwan; Division of Forest Protection, Taiwan Forestry Research Institute, Taipei, 10066 Taiwan; Research Center for Plant Medicine, National Taiwan University, Taipei, 10617 Taiwan

## Abstract

**Background:**

*Citrus exocortis viroid* (CEVd) and *Hop stunt viroid* (HSVd) are commonly found simultaneously infecting different citrus cultivars in Taiwan. A crucial question to be addressed is how accumulations of these two viroids affect each other in an infected plant. In this study, we investigated the relationship between the two viroids at macroscopic and microscopic levels.

**Methods:**

CEVd and HSVd titers were examined by real-time RT-PCR in 17 plants of two citrus cultivars (blood orange and Murcott mandarin) every 3 months (spring, summer, fall and winter) from 2011 to 2013. Three nonparametric tests (Spearman’s rank correlation coefficient, Kendall’s tau rank correlation coefficient and Hoeffding’s inequality) were performed to test the correlation between CEVd and HSVd. Cellular and subcellular localizations of the two viroids were detected by digoxigenin- and colloidal gold-labeled *in situ* hybridization using light and transmission electron microscopy.

**Results:**

The two viroids were unevenly distributed in four different types of citrus tissues (rootstock bark, roots, twig bark and leaves). Compared with blood orange, Murcott mandarin was generally more susceptible to CEVd and HSVd infection. Both viroids replicated and preferentially accumulated in the underground tissues of the two citrus cultivars. Except for blood orange at high temperatures, significant positive correlations were observed between the two viroids in specific tissues of both cultivars. Relative to concentrations under single-infection conditions, the CEVd population significantly increased under double infection during half of the 12 monitored seasons; in contrast, the population of HSVd significantly increased under double infection during only one season. At cellular/subcellular levels, the two viroids showed similar localization patterns in four tissues and the cells of these tissues in the two citrus cultivars.

**Conclusions:**

Our findings of titer enhancement, localization similarity, and lack of symptom aggravation under CEVd and HSVd double infection suggest that the two viroids have a positive relationship in citrus. The combination of molecular and cellular techniques used in this study provided evidence of titer correlation and localization of co-infecting viroids in the host. These methods may thus be useful tools for exploring viroid–viroid and viroid–host interactions.

## Background

Viroids, which are small, circular, single-stranded noncoding RNAs, are the smallest known agents infecting a broad range of plants. With a tiny genome size (246–401 nt) and simple structure, viroids do not encode proteins and must depend on host-encoded factors and enzymes for replication [1–5]. Viroids are classified into two families, Pospiviroidae and Avsunviroidae, based on their secondary structures and several biological features.

Mixed virus/viroid and viroid/viroid infections are common in field-grown plants, but only a few studies have addressed this phenomenon. A possible mechanism for virus/viroid interaction was uncovered in a study using viral-encoded silencing suppressors, where a titer of *Citrus dwarfing viroid* (CDVd) was enhanced by *Citrus tristeza virus* (CTV) in Mexican lime but not in Etrog citron. Interactions between these pathogens differed among host plant cultivars, indicating that such interactions are likely dictated by the host [6, 7]. In viroid/viroid interactions, multiple viroids in various citrus hosts show complicated antagonistic or synergistic relationships that lead to different symptoms, canopy volumes, fruit yields and commercial performance. No obvious physiological changes in citrus hosts have been observed in mixed infections of CEVd and HSVd [8, 9]. Although co-infection by the two viroids does not cause severe symptoms in citrus, their interaction is intriguing because of their high co-infection rate in the field and their identical biological properties in the same host.

Viroid distribution in plant tissues and cells is related to the replication sites and movement of the pathogen. Early studies using fluorescence *in situ* hybridization (FISH) and *in situ* hybridization in transmission electron microscopy (ISH-TEM) revealed that CEVd and *Coconut cadang cadang viroid* (CCCVd) were each localized in vascular tissue and in the nucleoli of mesophyll cells. CEVd was distributed within the entire nucleus; CCCVd was mostly concentrated in the nucleolus, but with some viroids present in the nucleoplasm [10]. A different localization pattern was detected for *Avocado sunblotch viroid* (ASBVd); through the application of digoxigenin (DIG)- or biotin-labeled RNA probes, this viroid was found to be distributed mostly in chloroplasts and, to a lesser extent, in cytoplasmic vacuoles [11, 12]. In addition, *Potato spindle tuber viroid* (PSTVd) was detected only in specific parts of mature flowers in tomato and *Nicotiana benthamiana* plants [13]. In petunia, PSTVd was delivered to the embryo through ovules or pollen during reproductive tissue development before embryogenesis [14]. Despite these reports, no studies have addressed the possible relationship and distribution of viroid pairs in mixed infections.

The objectives of this study were to assess the titer relationship of two viroids in a mixed infection and to investigate corresponding viroid distribution patterns and population changes in the host. To achieve our objectives, we analyzed samples of two citrus cultivars—blood orange (*Citrus sinensis* [L.] Osbeck ‘Moro’) and Murcott mandarin (*C. reticulata* Blanco ‘Murcott’)—co-infected with CEVd and HSVd and collected seasonally over 3 years.

## Results

### Distribution of viroids in four tissues of the two citrus cultivars

**Fig. 1:**
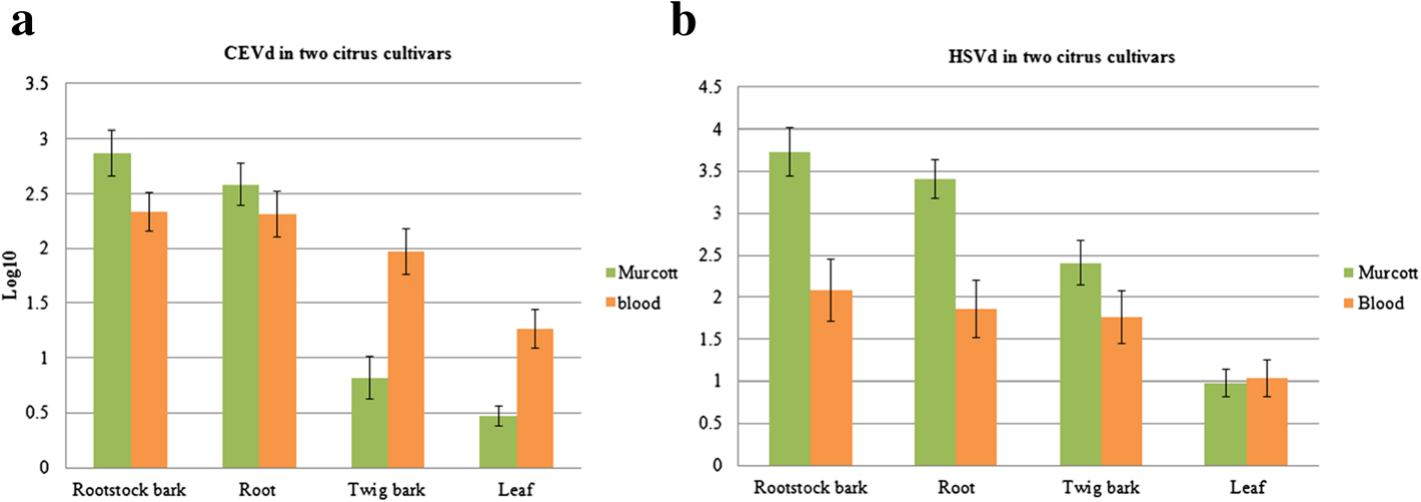
Titers of *Citrus exocortis viroid* (CEVd) and *Hop stunt viroid* (HSVd) in two citrus cultivars. Comparison of CEVd and HSVd quantities in four tissues of 15 blood oranges and Murcott mandarins by real-time RT-PCR. Copy numbers per microliter of (**a**) CEVd and (**b**) HSVd were analyzed in four tissues (rootstock bark, roots, twig bark and leaves) of two citrus cultivars. Data was averaged for nine blood oranges and six Murcott mandarins over 12 spring, summer, fall and winter 3-month periods, except for the data for the last two 3-month periods during which only two blood orange and five Murcott mandarin plants were available. Viroid copy numbers per microliter determined by real-time RT-PCR are displayed as log_10_ values. Bars represent standard deviation errors

During our 3-year survey, we observed the following distributions of the two viroids in 15 citrus trees: double infection of five blood oranges and four Murcott mandarins, CEVd single infection of two blood oranges, and HSVd single infection of two blood oranges and two Murcott mandarins. Two viroid-negative citrus plants were used as controls. CEVd accumulated more actively in roots and rootstock bark in both cultivars, but was obviously present in lower amounts in leaves and twig bark of Murcott mandarins. Although the two citrus cultivars had similar CEVd titers in roots (ca. 10^2.4^ RNA copies/μL) and rootstock bark (ca. 10^2.5^ RNA copies/μL), CEVd titers in twig bark and leaves of blood orange were nearly ten times higher than those in Murcott mandarin: 10^2^ vs. 10^0.8^ RNA copies/μL in twig bark and 10^1.3^ vs. 10^0.5^ RNA copies/μL in leaves. Murcott mandarin had higher HSVd titers in roots, rootstock bark and twig bark (10^3.4^, 10^3.7^ and 10^2.4^ RNA copies/μL, respectively) than did blood orange (10^1.9^, 10^2^ and 10^1.7^ RNA copies/μL, respectively) (Fig. 1). These data indicate that Murcott mandarin is generally more susceptible to CEVd and HSVd infection relative to blood orange. In the two citrus cultivars, both viroids replicate and preferentially accumulate in underground tissues.

### Population dynamics of CEVd and HSVd in all 17 studied citrus plants

**Fig. 2:**
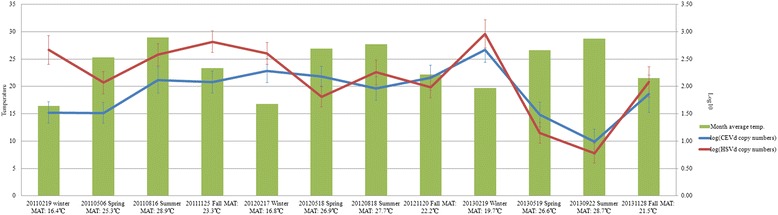
Seasonal dynamics analysis of *Citrus exocortis viroid* (CEVd) and *Hop stunt viroid* (HSVd) in citrus plants. Overview of 3-year population dynamics of CEVd and HSVd in 17 citrus plants from an infected field over 12 spring, summer, fall and winter 3-month periods. The statistical analysis incorporated 0.1 substitutions for under-determined values. The blue and red curves represent log_10_-transformed copy numbers of CEVd and HSVd, respectively. The blue and red bars indicate standard deviation errors. The green bars correspond to monthly average temperatures (MAT) during each 3-month period

A population dynamics analysis was conducted on all 17 plants, namely the 11 blood oranges and 6 Murcott mandarins under double-, single- or non-infection conditions as mentioned above. Monthly average temperatures were similar over the 3 years of the study and showed only a slight rise over the course of the experiment. The population size of HSVd was larger than that of CEVd during the first year; in subsequent years, however, the two viroids had similar population sizes (Fig. 2). CEVd titers increased steadily from the winter of 2011 through 2013, while HSVd titers generally followed the same pattern but decreased in 2012. The populations of both viroids obviously decreased in the spring and summer seasons of 2013. The titers of both viroids reached their highest concentrations of the 3-year study in the winter of 2013 (Fig. 2).

### Interaction of CEVd and HSVd titers

**Fig. 3:**
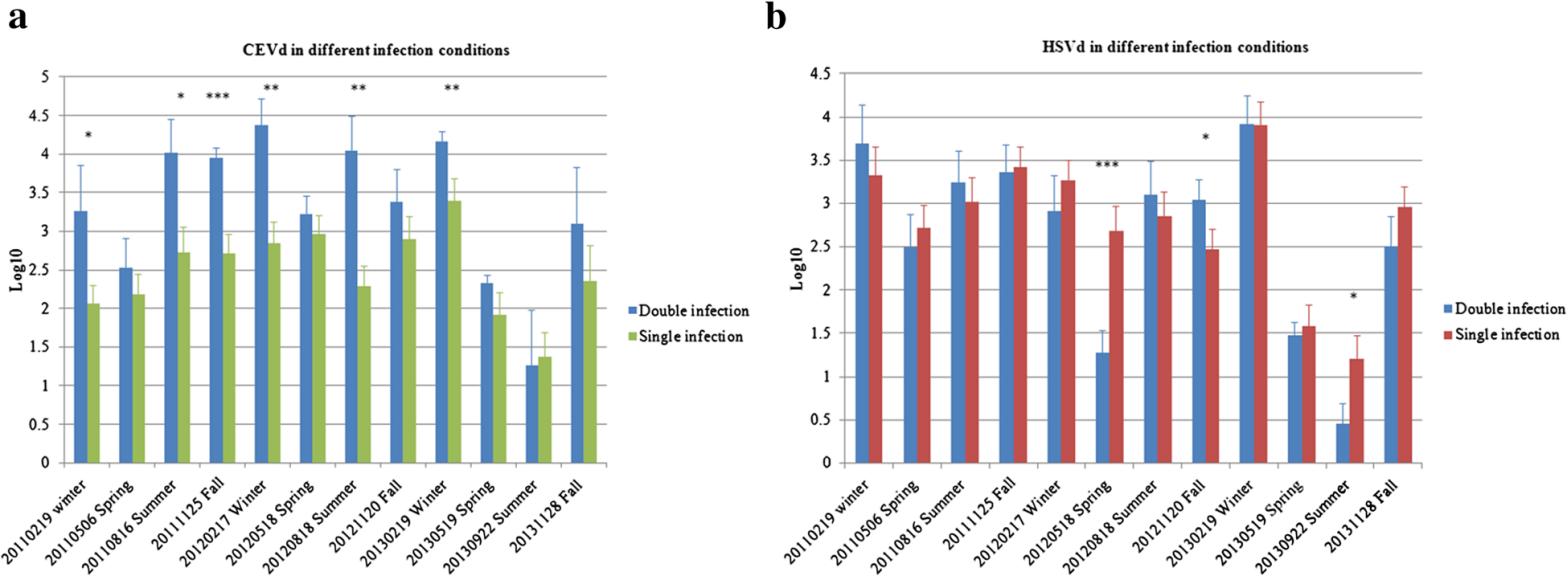
Comparison of populations of *Citrus exocortis viroid* (CEVd) and *Hop stunt viroid* (HSVd) under double- or single-infection conditions over 12 seasons. The doubly infected citrus trees consisted of five blood oranges and four Murcott mandarins. CEVd singly infected citrus trees comprised two blood oranges, and HSVd singly infected citrus trees were represented by two blood oranges and two Murcott mandarins. **a** CEVd populations under double- and single-infection conditions. **b** HSVd populations under double- and single-infection conditions. The y-axis corresponds to log_10_-transformed copy numbers of each viroid and the bars represent standard deviation errors. Statistically significant differences between values were determined by Student’s *t*-test (*P < 0.05; **P < 0.01; ***P < 0.001)

Titers of the two viroids were significantly positively correlated in all 15 infected citrus plants, both in the 6 singly infected citrus plants (with no under-determined data values) (*P* < 0.0001) and in the 9 doubly infected ones (*P* < 0.0004) (Table 1). The same results were obtained using 12 greenhouse-grown plants that were artificially doubly infected with CEVd and HSVd (Table 1). Titer levels of the two viroids were significantly positively correlated in rootstock bark and leaves of blood orange; a similar correlation in titer levels was observed in rootstock bark and roots of Murcott mandarin. Viroid titer levels were not correlated in any of the remaining tissues in either citrus cultivar. When temperature was factored into the analysis (Table 2), the two viroids showed strong positive correlations in Murcott mandarin at high, medium and low temperatures (*P* < 0.0001) and in blood orange at low and medium temperatures. In contrast, the two viroids were not correlated in blood orange under high temperature conditions. Significant correlations were generally observed in roots and rootstock bark of Murcott mandarins in all three temperature groups, whereas correlations were only observed in rootstock bark of blood oranges under medium temperatures. As determined by Student’s *t*-test (at *P* < 0.05, 0.01 or 0.001), populations of CEVd and HSVd under double-infection conditions showed statistically different levels of increase compared with populations of each viroid under single infection (Fig. 3). Compared with the CEVd population under single-infection conditions, the CEVd population under double infection increased significantly in size during half of the 12 monitored seasons (Fig. 3a). There was only one season in which the HSVd population under double infection increased significantly compared with the population under single infection (Fig. 3b).

**Table 1:** Significance of correlation coefficients for titers of *Citrus exocortis viroid* (CEVd) and *Hop stunt viroid* (HSVd) in four tissues of two citrus cultivars from the field and greenhouse

Correlation coefficients		
		CEVd and HSVd
Numbers of citrus plants	Tissues	Nonparametric methods^a^
17 citrus plants	All	++^b^
7 citrus plants^d^	All	++
11 Blood oranges	Roots	-^c^
	Rootstock bark	++
	Twig bark	-
	Leaves	++
6 Murcott mandarins	Roots	++
	Rootstock bark	++
	Twig bark	-
	Leaves	-
Double infections^e^	Roots	++
	Rootstock bark	++
	Twig bark	++
	Leaves	++
12 citrus plants^f^ (Sets in greenhouse)	All	++

^a^Three nonparametric methods are Spearman’s nonparametric correlation coefficient, Kendall tau rank correlation coefficient and Hoeffding’s inequality

^b^correlation coefficients marked with “++” are statistically significant by all three methods; those marked with “+” are statistically significant by two of three methods

^c^indicates no significant difference

^d^There were no underdetermined values in this data set including 3 Blood oranges and 4 Murcott mandarins

^e^includes 5 Blood oranges and 4 Murcott mandarins

^f^Artificially double inoculated plants include 6 Blood oranges and 6 Murcott mandarins

**Table 2:** The significance of correlation coefficients between *Citrus exocortis viroid* (CEVd) and *Hop stunt viroid* (HSVd) from two infected citrus cultivars under different temperature conditions

	Correlation coefficients between CEVd and HSVd					
	Blood orange^a^			Murcott mandarin^a^		
Temperature	Low	Moderate	High	Low	Moderate	High
All^b^	++^c^	++	-	++	++	++
Rootstock bark	-^d^	++	-	++	+	++
Root	-	-	-	-	++	++
Twig bark	-	-	-	-	-	-
Leaf	-	-	-	-	++	-

^a^Three nonparametric methods are Spearman’s nonparametric correlation coefficient, Kendall tau rank correlation coefficient and Hoeffding’s inequality

^b^All = overall data of blood oranges or Murcott mandarins

^c^correlation coefficients marked with “++” are statistically significant by all three methods and those marked with “+” are statistically significant by two of three methods

^d^indicates data are not significantly difference

### Tissue distributions of the two co-infected viroids assessed by DIG-labeled *in situ* hybridization (DIG-ISH)

**Fig. 4:**
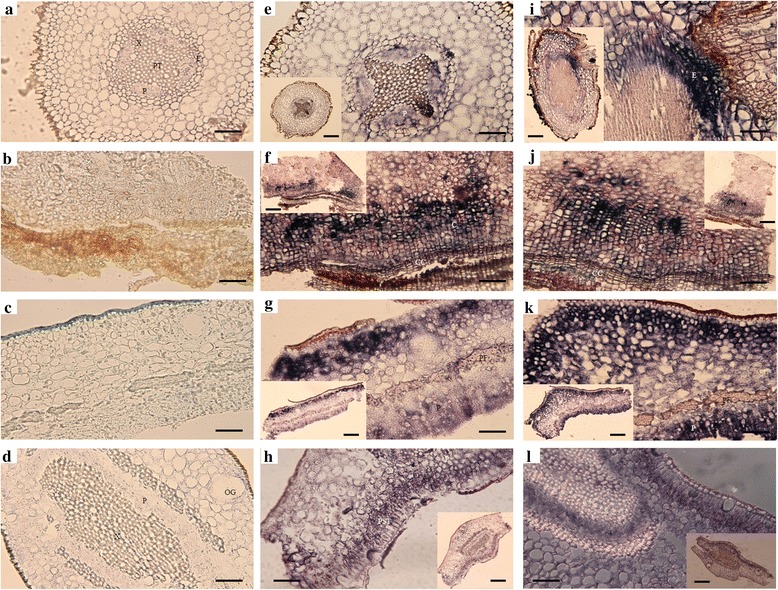
Tissue distributions of *Citrus exocortis viroid* (CEVd) and *Hop stunt viroid* (HSVd) revealed by digoxigenin-labeled *in situ* hybridization (DIG-ISH) under a transmitted light microscope. **a**-**d**) Viroid-negative citrus tissues hybridized with viroid DIG-labeled probes as controls. **e**-**h**) Co-infected citrus tissues hybridized with CEVd DIG-labeled probes. **i**-**l**) Co-infected citrus tissues hybridized with HSVd DIG-labeled probes. **a**, **e** and **i** Transverse sections of viroid-negative (**a**) and co-infected (**e** and **i**) citrus roots. **b**, **f** and **j** Transverse sections of viroid-negative (**b**) and co-infected (**f** and **j**) citrus rootstock bark. **c**, **g** and **k**) Transverse sections of viroid-negative (**c**) and co-infected (**g** and **k**) citrus twig bark. **d**, **h** and **l**) Transverse sections of viroid-negative (**d**) and co-infected (**h** and **l**) citrus leaves. Bars in original photographs (**a**-**l**) = 5 μM; bars in small photographs (**e**-**l**) = 15 μM. PT = pith; P = phloem; X = xylem; E = endodermis; C = cortex; CC = cork cambium; PF = phloem fiber; OG = oil gland; PST = palisade tissue

To evaluate the distributions of the two positively correlated viroids in four citrus tissues, DIG-labeled riboprobes of CEVd and HSVd were separately used for detection. We observed that the two viroids occupied similar locations in continuous sections of the four examined tissues. In root tissues, both viroids localized mostly in the endodermis and pericycle (Fig. 4e and i). In rootstock bark tissues, the viroids were detected in cortical cells near the cork cambium (Fig. 4f and j). Both viroids occupied outer cortical and phloem cells in twig bark tissues (Fig. 4g and k) and were localized in palisade tissues and phloem cells in leaf tissues (Fig. 4h and l).

### Subcellular distributions of the two co-infecting viroids determined by multiplex colloidal gold-labeled *in situ* hybridization (ISH-TEM)

**Fig. 5:**
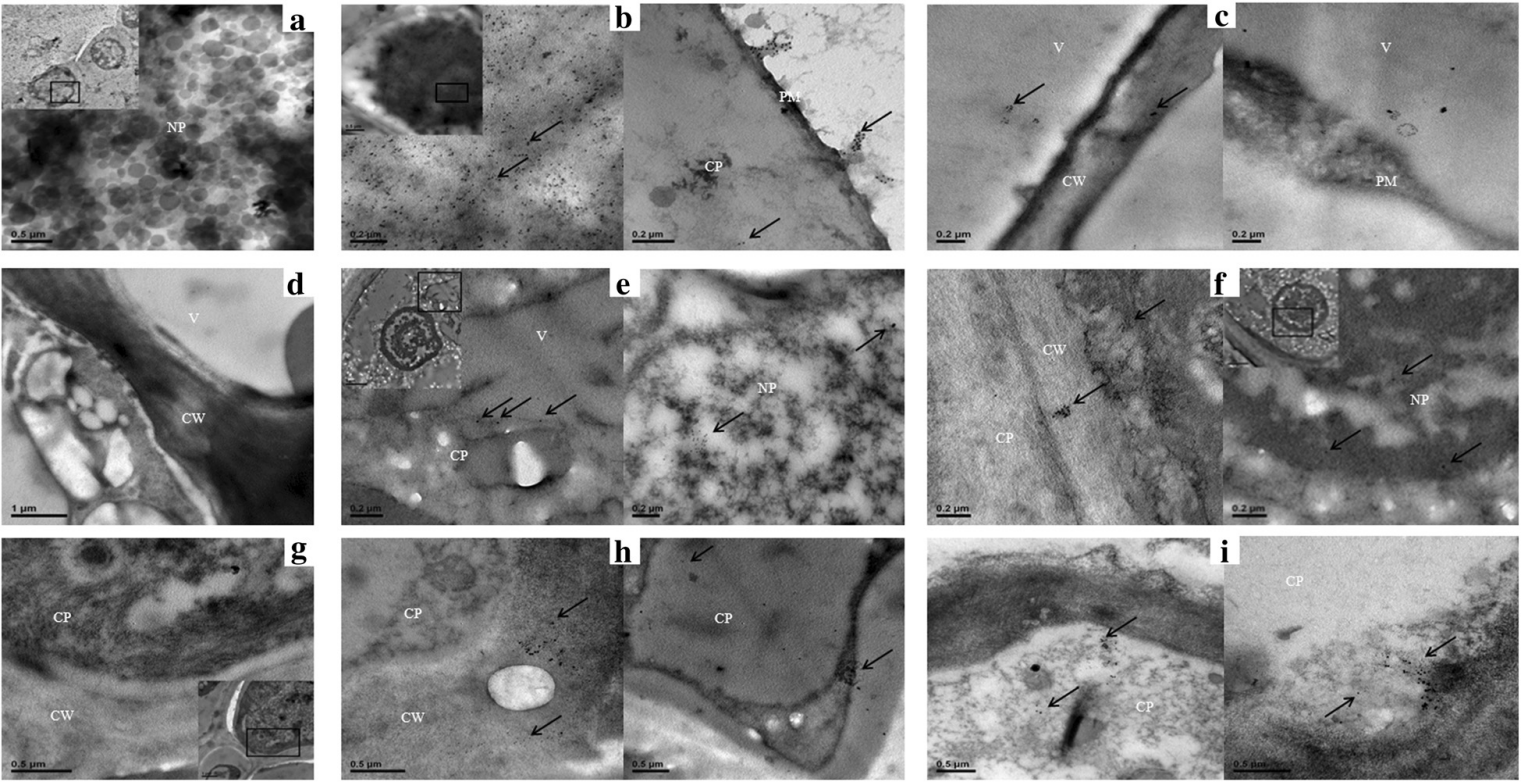
Transmission electron micrographs showing subcellular localization of *Citrus exocortis viroid* (CEVd) and *Hop stunt viroid* (HSVd) in two citrus cultivars. The locations of CEVd and HSVd in three tissues of blood oranges and Murcott mandarins were detected by *in situ* hybridization with digoxigenin (DIG)-labeled and biotinylated anti-sense riboprobes, respectively. The locations of CEVd DIG-labeled probes were detected by anti-DIG monoclonal antibody as a primary antibody and a 10-nm diameter colloidal gold conjugate of Alexa Fluor 488 goat anti-rabbit IgG as a secondary antibody. HSVd biotin-labeled probes were detected with 20-nm colloidal streptavidin-gold from *Streptomyces avidinii*. Ultrathin sections of viroid-negative roots (**a**), rootstock bark (**d**) and twig bark (**g**) hybridized with the same probes revealed neither CEVd nor HSVd signals in the nucleus nor in any other subcellular structures. **b** Ultrathin sections of mature blood orange roots infected by the two viroids. Most of the probe signals were associated with the nucleus and present near the plasma membrane and cytoplasm. **c** Ultrathin sections of mature roots of co-infected Murcott mandarin. The viroid signals were associated with the vacuole and cell wall. **e** Ultrathin sections of rootstock bark of co-infected blood orange. The probes were associated with the nucleoplasm or cytoplasm. **f** Ultrathin sections of rootstock bark of co-infected Murcott mandarins. The viroid signals were associated with cell walls, cytoplasm and the nucleoplasm. **h** Ultrathin sections of twig bark of co-infected blood orange. The probes were associated with the cytoplasm and cell walls. **i** Ultrathin sections of twig bark of co-infected Murcott mandarins. The viroid signals were associated with the cytoplasm. NP = nucleoplasm; V = vacuole; PM = plasma membrane; CP = cytoplasm; CW = cell wall

Three citrus tissues with high viroid titers (roots, rootstock bark and twig bark) were used to evaluate subcellular distributions of the two location-correlated viroids. CEVd and HSVd riboprobes were labeled with DIG and biotin, respectively, as antigens for antibody detection. We used 10- and 20-nm diameter colloidal gold particles to respectively differentiate the localizations of CEVd and HSVd RNAs. Our analysis revealed that the two viroids were generally present in the nucleoplasm, vacuoles, cytoplasm, plasma membranes and cell walls of all three tissues. In roots, the two viroids were mostly present in the vacuoles and nucleoplasm (Fig. 5b and c). In Murcott mandarins, a few HSVd signals could be found in cell walls, with CEVd signals detected in the vacuoles. In rootstock bark, both viroids were more densely distributed in the nucleoplasm, with HSVd found in the cell walls but not in the vacuoles (Fig. 5e and f). In twig bark, massively intense HSVd signals were observed in the cytoplasm; similarly, CEVd could also be detected in the cytoplasm (Fig. 5h and i). No obvious difference in viroid subcellular localization was found between the two citrus cultivars. Both viroids were concentrated mostly in the nucleoplasm, vacuoles and cytoplasm rather than in the other subcellular compartments.

## Discussion

We have previously surveyed blood oranges and Murcott mandarins in Taiwan and confirmed the frequent occurrence of simultaneous CEVd and HSVd infections [15]. The infected hosts exhibited only typical exocortis symptoms, thus increasing the difficulty of measuring the interaction between the two viroids. In addition, previous research has detailed the distributions of viroids in host plants [10–12], but the association between symptom expression and viroid location is unclear. In this study, we therefore used molecular, statistical and *in situ* hybridization methods to look for correlations between the two viroids and to determine the relationship between their interaction and distribution.

According to our seasonal investigation of the two citrus cultivars grafted onto susceptible Rangpur lime rootstock, viroid titers were higher in underground parts than in aerial portions, and the titer of HSVd was also higher than that of CEVd. In general, viroid populations ranged from 10^2^ to 10^4^ copy numbers per microliter of total RNA extract. Murcott mandarins were more susceptible than blood oranges to infection. Accumulations of HSVd were nearly 10-fold higher than those of CEVd in Murcott mandarins; this suggests that unknown factors in Murcott mandarin contributed to the higher accumulated titers, even though the two viroids likely use the same replication mechanism. The titer of HSVd in Murcott mandarins was almost doubles that in blood oranges, indicating different interactions between this viroid and the two citrus cultivars. The reason for the obvious decrease in CEVd titers in twig bark and leaves of Murcott mandarins is not clear. Additional research may help explain the uneven distributions in different tissues, and, in particular, why the viroids could barely be detected in leaves. With the complete sequencing of the draft genome of sweet orange [16], the interaction between a citrus host and its viroid pathogens may be further studied at the molecular level.

Whether antagonistic or synergistic, the interaction between two viroids depends on multiple factors, including pathogenicity, host susceptibility, and competition for resources. The defined nature of the interaction between two plant viruses depends on the extent of symptoms and pathogen titers [17]; this is probably true for viroids as well. Previous studies have demonstrated that viroid co-infections affect a host plant’s physiology in various ways, including delayed flowering [18], yield reduction, symptom aggravation, canopy rarefaction and dwarfing [6, 8, 9]. The HSVd isolates in Taiwan were non-cachexia variants, but with no differences observed in exocortis symptoms of co-infected citrus plants compared with those infected only with CEVd. A previous long-term investigation carried out from 1996 to 2001 similarly revealed no significant differences in yields or other growth parameters when CEVd and HSVd co-infected Commune clementine grafted onto Pomeroy trifoliate orange [8]. As a consequence, dynamic titers are by necessity the major indicators available to confirm viroid interactions in citrus. As shown in Fig. 3, CEVd titers under co-infection conditions were more significantly enhanced than those of HSVd during six sampling seasons in which their titers were statistically strongly correlated. We speculate that this difference is due to the small number of doubly infected citrus plants sampled. To provide firmer evidence for CEVd/HSVd interaction, additional samples should be added in future experiments. Furthermore, various combinations of mild and severe CEVd and HSVd isolates should be considered to test for different degrees of interaction among them. Although our data did not reveal a statistically significant synergistic interaction between the two viroids on the basis of correlation analyses and Student’s *t*-test, we nonetheless conclude that CEVd and HSVd may have a positive relationship—as evidenced by titer enhancement, localization similarity, and lack of symptom aggravation under double infection in citrus.

Surprisingly, CEVd and HSVd do not appear to compete for resources even though they are both in the family Pospiviroidae and have many biological properties in common. This situation is counter-intuitive because similar pathogens usually compete for the same resources when colonizing the same space. We were unable to discern the mechanism of interaction between the two viroids at macroscopic or microscopic levels. A previous study, in which titers of *Citrus dwarfing viroid* (CDVd) were enhanced by the co-infecting *Citrus tristeza virus* (CTV) in Mexican lime, provides a possible clue: the CTV RNA silencing suppressor p23 aided CDVd invasion and replication [7]. Our results suggest that temperature might be another factor affecting the interaction between the two viroids (Table 2). To explain this phenomenon, we hypothesize that CEVd and HSVd use different host cell resources under specific temperature conditions to fulfill their biological functions. Alternatively, each viroid may rely on an unknown mechanism to interact with the other one in a positive fashion. Additional research involving proteomic or transcriptomic analysis of viroid co-infected citrus should be carried out under different temperature conditions [19, 20].

Previous studies have shown that viroids in the family Pospiviroidae replicate in the nucleus, are distributed to other organelles, and spread via phloem, resulting in systemic infection [11]. However, no studies have focused on the distributions of interacting viroids and whether their distributions are complimentary or antagonistic. Our investigation has confirmed that CEVd and HSVd are both concentrated in endodermal cells and the pericycle of root tissues. In a previous study in tomato, PSTVd was similarly detected in the outer part of the central cylinder containing the endodermis, pericycle and vascular tissue [21]. Our study is the first to identify the location of two viroids in bark exhibiting classical exocortis symptoms. We have shown that the two viroids are concentrated only in cortical cells near the cork cambium and not in inner cortical cells. We postulate that the specific distributions of the viroids may be related to an unknown pathogenicity mechanism that controls exocortis formation. To study subcellular-level interactions between the two viroids, we used the ISH-TEM method to simultaneously detect CEVd and HSVd localizations. To our knowledge, our study is the first to simultaneously detect a pair of interacting viroids in two citrus cultivars.

## Conclusions

This study focused on the interaction between CEVd and HSVd at both macroscopic and microscopic levels. Statistically supported positive correlations were uncovered between the two viroids in rootstock bark and leaves of blood oranges and in roots and rootstock bark of Murcott mandarins. The two viroids accumulated according to similar patterns in four citrus tissues at cellular/subcellular levels. Future research should focus on why two viroids in Pospiviroidae with similar biological functions and sharing identical cellular and subcellular spaces do not appear to compete with one another for host resources.

## Materials and methods

### Seasonal collection of plant materials and viroid sources

Based on our previous field survey, the rectangle field we chose, in Yunlin County, Taiwan, was 5 m × 500 m in size (the coordinate of the field is 23°41′53.6″N 120°35′29.6″E). The study was carried out on private land; the owner of the land gave permission to conduct the study on this site. No specific permissions were required for these locations/activities and we confirmed that the field studies did not involve endangered or protected species. The rootstocks used in this field were all of 30 to 35 years old susceptible cultivars Rangpur limes (*Citrus limonia* Osbeck). Samples of about 10–100 g were collected from each of four tissues (root, rootstock bark, twig bark and leaf) from 11 randomly chosen blood oranges (*Citrus sinensis* (L.) Osbeck cv. “Moro”) and 6 murcott mandarins (*Citrus reticulate* Blanco cv. ‘Murcott’), which were uninfected as viroid-negative controls or infected by *Citrus exocortis viroid* (CEVd) or *Hop stunt viroid* (HSVd) (non-cachexia variant). The sampling criteria were as follows: For the root, we chose the slim branch roots about a few centimeters long; for rootstock bark, we skived the barks, about 5–10 cm above the ground, which were 2 cm long, 5 cm wide and 0.2 cm thick for each; for twig bark and leaf, young or mature twigs with leaves were cut in 10 cm for each, and the twig barks were torn and the xylem was excluded by tweezers. For each tissue type, samples were collected from three positions of a single citrus tree to ensure random sampling. We collected the samples consistently every three months from February, 2011 to November, 2013. To conduct the relationship analysis of correlation test in green house, 15 cm tall citrus plants were artificially inoculated with the two viroids. For correlation analysis, the conditions of double- or single- or non- infection of two viroids were conducted at 25 °C in green house. Each test repeated at least three bio replicates.

### RNA extraction and cDNA amplification

Viroid RNAs were extracted by several modifications of the TRIzol RNA isolation method [22]. A citrus tissue sample (150 mg) was homogenized in 1.5 mL TRIzol reagent (0.8 M guanidine thiocyanate; 0.4 M ammonium thiocyanate; 0.1 M sodium acetate, pH 5.0; 5 % glycerol; 38 % phenol in saturated buffer). The supernatant was collected after centrifugation (13,500 x *g*) for 10 min and, after a 1/5 volume of chloroform was added, was vortexed vigorously for 15 s and placed at room temperature for 3 min. The supernatant was collected. A 1/2 volume of isopropanol and 1/2 volume of 0.8 M sodium citrate/1.2 M NaCl were added and then incubated at room temperature for 10 min. The RNA pellet was collected by high speed (17,000 x *g*) centrifugation at 4 °C for 15 min. The pellet was washed with 70 % ethanol and resuspended in DEPC-water to a final concentration of 300 ng/μL. cDNA was prepared by M-MLV Reverse Transcriptase (Gibco BRL) according to the manufacturer’s instructions. The total volume was 20 μL containing 50 units of M-MLV reverse transcriptase, 50 mM Tris–HCl (pH 8.3), 75 mM KCl, 3 mM MgCl_2_, 10 mM DTT, 0.5 mM dNTP, 2 pmol reverse primer and 2 μg template of nucleic acid. The reaction mixture was mixed gently and incubated at 42 °C for 50 min. The mixture then was diluted to 15 ng/μL for real-time PCR.

### Real-time PCR

For absolute quantification of CEVd and HSVd titers, quantitative real-time PCR amplification of CEVd and HSVd was conducted as described by our previous study [15]. Briefly, for quantitative real-time PCR amplification of CEVd and HSVd cDNAs: CEVd-RTR_F (5′-GTCGCCGCGGATCACT-3′), CEVd-RTR_R (5′-CCAGCAGCGAAAGGAAGGA-3′) and CEVd-RTR_P (5′-CCAGCGGAGAAACAG-3′) leading to a product of 64 bp of CEVd; HSVd-RTR_F (5′-GGAATTCTCGAGTTGCCGCA-3′), HSVd-RTR_R (5′-CCGCGGCCCTCTCT-3′) and HSVd-RTR_P (5′-CAACTCTTCTCAGAATCC-3′) leading to a product of 127 bp of HSVd. The 5′ terminal reporter dye of probe was FAM (6-carboxyfluorescein), and the 3′ quencher dyes were NFQ (non-fluorescent quencher) and MGB (minor groove binder). The TaqMan PCR reactions (total volume 20 μL) were set up in 96-well reaction plates using PCR mastermix reagent kits (Applied Biosystems). The real-time PCR conditions followed Applied Biosystems’ instruction with slight modifications:6.35 μL (75 ~ 95 ng) of cDNA, 10 μL 2X Taqman^®^ Gene Expression Master Mix, 3.6 μL primer set (900nM forward primer and reverse primer), 0.05 μL (250nM Taqman^®^ MGB probes), and ROX passive reference dye (concentration undisclosed by manufacturer). The assays were operated on an ABI StepOne Real-Time PCR System (Applied Biosystems) at cycling conditions (50 °C/2 min, 95 °C/10 min and 40 cycles of 95 °C/15 min, 60 °C/1 min).

### Statistical analysis

To determine viroid titers by real-time RT-PCR, the Ct values of each viroid sample ranged from 16.5 to 33, which corresponded to initial viroid titers from 10^7^ to 10^2^ copy numbers per microliter, respectively. The analyzed data contained many underdetermined values, which meant that the Ct values were up to 36 and apparently viroid-free, as well as the viroid-negative control that also was viroid-free. To transform the exponents of copy numbers into numbers by Log10, the underdetermined values became deviations in statistical analysis. Therefore, any underdetermined values were assigned values of 0.1 in the data set. Furthermore, the collected samples were incomplete in the last three 3-month periods because the grower removed several plants. A total of 1,356 individual data values from two viroids in the two citrus cultivars in twelve 3-month periods over 3 years were analyzed by using JMP7.0 (SAS Institute Inc.). Data were first grouped into three categories depending on high (summer, 27.7–28.9 °C), moderate (spring and fall, 21.5–26.9 °C) and low (winter, 16.4–19.7 °C) temperatures. The following analysis was based on a previous reference [23]. First, the total individual data were separated into two viroid groups and evaluated to determine whether the two groups were normally distributed. The hypothesis of normal distribution was rejected because the *P*-value was lower than 0.05 through the Goodness-of-fit test using the Shapiro-Wilk W test. Then the difference between cultivars or infection situations among categories was evaluated through three nonparametric tests (Spearman’s rank correlation coefficient; Kendall tau rank correlation coefficient and Hoeffding’s inequality) to test the correlation between CEVd and HSVd. If the analyzed *P*-value was less than 0.05, then the two factors had a positive correlation coefficient.

### *In situ* hybridization with transmitted light microscopy

#### Specimen preparation

Sample processing followed existing protocols [13, 24] with modifications. Four parts of tissues from viroid-negative and viroid-infected citrus were cut continuously into strips of approximately 1 cm × 3 mm and fixed overnight in FAA at 4 °C, followed by dehydration through an ethanol dilution series and xylene at room temperature. Once in pure xylene, 20 chips of paraplast were added to the sample vials. The vials were placed at 42 °C for 2 h until paraplast was completely dissolved. After two more additions of the same amounts of paraplast, the xylene/paraplast mixture was replaced with fresh molten paraplast and the vials were placed at 60 °C overnight. The paraffin-infiltrated samples were embedded in embedding cassettes by using Leica EG1150 Embedding Center following the manufacture’s protocol (Leica biosystems, St Louis, MO, USA). Paraffin sections of 10 μm thicknesses were obtained with a Leica RM2235 Microtome (Leica biosystems, St Louis, MO, USA) and kept on aminoalkysilane coated slides at 4 °C for preservation.

#### DIG-labeled probe preparation and purification

Viroid transcripts of 302 ~ 372 nt containing positive monomeric full-length sequences of CEVd or HSVd were cloned into pcr2.1-TOPO vectors (Invitrogen, Life technologies, Carlsbad, CA, USA) as templates to allow generation of complimentary-strand probes (CEVd-p; HSV-p). *In vitro* transcription with T7 polymerase and DIG-modified UTP (DIG-11-UTP) was performed following the manufacturer’s protocol (Roche, Basel, Switzerland). The transcripts (20 μL) were diluted with nuclease-free water and then purified by centrifugation with phenol/chloroform. The purified transcripts were precipitated by addition of 1/10 volume of 3 M sodium citrate (pH5.2), 1/100 volume of glycogen and 3 volume of 100 % ethanol followed by centrifugation at 17,000 g for 15 min. The pellets were resuspended in 5 μL of nuclease-free water. Normally 1 μg of viroid-contained plasmid could generate approximately 3–5 μg of DIG-labeled RNA probe in our study. DIG-labeled probes were stored at −80 °C.

#### DIG-labeled *in situ* hybridization

The slides containing paraffin sections were dewaxed by immersion in xylene for 4 min and then 100 % ethanol for 4 min twice. After hydration through a graded ethanol dilution series from 95 to 30 % and then distilled water, the slides were treated with proteinase K in TE buffer (100 mM Tris–HCl, 50 mM EDTA, pH 8.0) at 37 °C. Next, the specimens were acetylated with 0.5 % acetic anhydride in 0.1 M triethanolamine (TEA) buffer (pH 8.0) for 10 min at room temperature. The specimens were then incubated in hybridization buffer (50 % formamide, 20X SSC, 100 μg/mL fragmented salmon testes DNA, 5 M NaCl, 0.5 M EDTA) containing viroid probes at 58 °C for 20 ~ 48 h. After the hybridization step, the slides were washed twice in 2X SSC at room temperature for 20 min and once in 0.2X SSC at 55 °C for 30 min. Afterwards the slides were incubated with alkaline phosphatase-conjugated anti-DIG antibodies (1:750 dilution in a buffer of 100 mM Tris of pH 7.5, 150 mM NaCl, 0.3 % Triton X-100, and 1 % bovine serum albumin) for 3 h at room temperature. After a final wash with buffer 1 (100 mM Tris–HCl and 150 mM NaCl) (pH 7.5), the specimens were incubated with the color substrate solution (100 mL of NBT/BCIP in 5 mL of AP buffer) in the dark. When color had developed sufficiently, the specimens were mounted and examined under System Microscope Olympus BX53 (Olympus Corporation, Tokyo, Japan).

### *In situ* hybridization with transmission electron microscopy

#### Specimen preparation

The procedure of *in situ* hybridization in TEM followed the description in a protocol book [25]. Six co-infected citrus trees were selected and prepared for transmission electron microscopy. Four tissues of viroid-infected and viroid-negative citrus plants were cut into strips of approximately 0.3 cm × 0.1 cm and then prefixed in a mixture of 0.25 % glutaraldehyde (GA) and 4 % paraformaldehyde (PFA) in 0.1 M phosphate buffer (0.1 M Na_2_HPO_4_, 0.1 M NaH_2_PO_4_, pH 7.4) at 4 °C for four hours, washed with 0.1 M phosphate buffer for fifteen minutes (three times), dehydrated through an ethanol dilution series from 30%, 50%, 70%, 90% and finally to 100 % for 30 min each, repeating the last step twice. Afterwards the samples were infiltrated with three mixture ratios (3:1, 1:1, 1:3) of 100 % ethanol and medium grade LR White low viscosity resin (London Resin Company, Reading, Berkshire, UK) overnight (twice and 4 h for the last step) and then with 100 % LR White resin overnight and repeated for 4 h once at room temperature. The samples were finally embedded in pure LR White resin which was cured for 48 h at 60 °C. Sections of 90 nm were obtained with a diamond knife on an RMC PT-X PowerTome Ultramicrotome.

#### Colloidal gold labeled probe preparation and purification

Here we chose digoxigenin (DIG) and biotin as tags on riboprobes for multiplex *in situ* hybridization in TEM. The positive strand, monomeric full-length sequences of CEVd and HSVd were cloned into pcr2.1-TOPO vectors (Invitrogen™, Life technologies, Carlsbad, CA, USA) as templates for generating single-stranded RNA probes by *in vitro* transcription. *In vitro* transcription with T7 polymerase incorporating digoxigenin-11-UTP or biotin-11-UTP were performed as follows. For CEVd, the riboprobe with DIG labeled system was generated following the instructions of the DIG Northern Starter Kit (Roche, Basel, Switzerland). The total volume was 10 μL containing 1 μg linearized plasmids, 5X transcription buffer, 5X labeling mix with digoxigenin-11-UTP and T7 polymerase. The mixture was centrifuged briefly and incubated for 1 h at 42 °C. DNase I was added for 15 min at 37 °C to remove the DNA templates. The DIG-labeled probes were purified by PI/PCI precipitation method as mentioned above. For HSVd, the riboprobe was produced by following the instructions of the mMESSAGE mMACHINE^®^ Kit (Ambion^®^, Life Technologies, Carlsbad, California, U.S.A.). The total volume was 20 μL containing 1 μg linearized plasmids, 2X NTP/CAP (12 mM cap analog, 3 mM GTP and 15 mM ATP, CTP and UTP with substitution to 15 mM biotin-11-UTP), 10X reaction buffer, and T7 polymerase. The mixture was incubated for 1 h at 37 °C. TURBO DNase was added and incubated for 15 min at 37 °C. The biotin-labeled probes were purified by the same precipitation protocol as above. Both probes were stored for 1 month at −80 °C.

#### Multiplex *in situ* hybridization with colloidal gold labeled and transmission electron microscopy

The *in situ* hybridization included five steps: pretreatment, hybridization, post-hybridization, immuno-cytochemical visualization, and staining. For pretreatment, the grids containing tissue sections were first treated with proteinase K in Tris/CaCl_2_ for 15 min at 37 °C and then washed with Tris/CaCl_2_ and phosphate buffer for 5 min each. The sections were then post-fixed with 2.5 % GA in PB buffer for 5 min before washing with PB buffer for three times. The grids were prewashed in Tris/MgCl_2_ buffer for 10 min and then digested by DNase I for 1 h, followed by washing with sterile water for 2 min (three times). The grids were treated with prehybridization buffer (1/2 volume of deionized formamide, 0.25 M NaHPO_4_, 0.25 M NaCl, 1 mM EDTA, 10 mg/ml salmon sperm DNA and 4X SSC) for 2 h at room temperature, followed by further treatment with 0.5 N NaOH to expose the sample surface for 4 min. After three washes with sterile water, the grids were air dried.

#### Hybridization

The probes were denatured for 5 min at 90 °C and the concentrations of the probes were between 10 to 20 μg/mL. The samples were incubated with hybridization buffer containing the probes at 37 °C for overnight.

#### Post-hybridization

The grids were sequentially washed with a mixture of 30 % formamide and 4X SSC buffer for 5 min, 4X SSC for 10 min (twice), and 2X SSC buffer for 5 min (twice).

#### Immuno-cytochemical visualization

2.5 % GA in PB buffer were used to stabilize the sections on the grids for 5 min before washing with PB buffer for 5 min (three times). The grids were then treated with phosphate/NaCl buffer with 1 % BSA for 30 min before incubation with anti-digoxigenin monoclonal antibody (clone 9H27L19) and ABfinity™ Recombinant (0.5 μg/mL) (Invitrogen™, Life technologies, Carlsbad, CA, USA) in phosphate/NaCl buffer for 1 h at room temperature. The grids were washed with PN buffer and Tris–HCl/NaCl buffer for 5 min, twice for each, and treated for 1 h with a mixture of Alexa Fluor® 488 goat anti-rabbit IgG, 10 nm colloidal gold conjugate (0.3 μg/mL) (Invitrogen™, Life technologies, Carlsbad, CA, USA) and streptavidin − gold from *Streptomyces avidinii*, and 20 nm colloidal gold (0.5 μg/mL) (Sigma-Aldrich Corporation, St. Louis, MO, USA) in Tris–HCl/NaCl buffer. After Tris–HCl/NaCl buffer and 2X SSC washes for 5 min, twice for each, the grids were treated with 2.5 % GA in PB buffer for 5 min and washed with 2X SSC and sterile water for 5 min each.

#### Staining

The grids were treated with 6 % uranyl acetate (UA) for 20 min and then washed with sterile water for 4 min (5 times). The grids were then air dried for 15 min before observation in a Hitachi H-7650 microscope at 75 kV.
